# Epidemiology of respiratory pathogens in patients with acute respiratory infections during the COVID‐19 pandemic and after easing of COVID‐19 restrictions

**DOI:** 10.1128/spectrum.01161-24

**Published:** 2024-09-25

**Authors:** Pei Zhao, Yu Zhang, Jie Wang, Yonghui Li, Yuxin Wang, Yuan Gao, Mengchuan Zhao, Ming Zhao, He Tan, Yanqing Tie, ZhiShan Feng

**Affiliations:** 1 Department of Clinical Laboratory Diagnosis, Hebei Medical University, Shijiazhuang, Hebei, China; 2 Department of Clinical Laboratory, Hebei General Hospital, Shijiazhuang, Hebei, China; 3 Hebei Center for Disease Control and Prevention, Shijiazhuang, China; 4 Graduate School, Hebei Medical University, Shijiazhuang, Hebei, China; 5 Hebei Key Laboratory of Molecular Medicine, Shijiazhuang, China; 6 Hebei Clinical Research Center for Laboratory Medicine, Shijiazhuang, China; Children's National Hospital, George Washington University, Washington, DC, USA

**Keywords:** COVID‐19 pandemic, epidemiology, acute respiratory infections, respiratory pathogens

## Abstract

We aimed to investigate the epidemiological characteristics of non-severe acute respiratory syndrome coronavirus 2 (SARS-CoV-2) respiratory pathogens among patients with acute respiratory infections (ARIs) in Shijiazhuang, China, during the coronavirus disease 2019 (COVID‐19) pandemic (January 2021-–December 2022) and after the relaxation of COVID‐19 restrictive measures (January 2022-–December 2023). This retrospective study enrolled 6,633 ARIs patients who visited the Hebei General Hospital between 2021- and 2023. Nasopharyngeal swabs samples were collected for multiplex PCR detection of 13 common respiratory pathogens. Respiratory pathogens were detected in 31.58% of individuals diagnosed with ARIs, whileereas a co-infection with multiple pathogens was observed in 8.5% of the ARI patients. In the years 2021 and 2022, 326 (27.63%) and 283 (24.38%) respiratory pathogens were found to be positive, respectively, during the COVID-19 pandemic. However, in 2023, subsequent to the easing of COVID-19 restrictions, the positivity rate significantly rose to 34.62%, with 4,292 cases identified. The majority of positive cases over the last three3 years were concentrated in patients under 14 years old. The predominant pathogens identified were human rhinovirus (HRVs) (15.08%) in 2021, mycoplasma pneumonia (MP) (6.46%) in 2022, and influenza A virus (FluA) (11.35%) in 2023. Seasonal prevalence patterns of most pathogens were affected, except for parainfluenza virus (PIV). There was a simultaneous increase in the positive cases and positivity rates of FluA and adenovirus (ADV) Iin 2023, compared to 2021 and 2022. Additionally, the infection rates of respiratory syncytial virus (RSV), MP, and coronavirus (CoV) in 2023 either exceeded or were comparable to those in 2021 and 2022. Conversely, the positivity rates of PIV, RVs, metapneumovirus (MPV), and influenza B virus (FluB) were lower in 2023 compared to 2021 or 2022.

**IMPORTANCE:**

The implementation of strict non-pharmaceutical interventions (NPIs) during the coronavirus disease 2019 (COVID-19) pandemic may lead to changes in the epidemiological features of respiratory pathogens, as well as the occurrence of immune debt, potentially causing a resurgence in respiratory pathogen activity following the easing of strict NPIs measures. There are limited reports on the epidemiological characteristics of respiratory pathogens among patients of all ages with acute respiratory infections (ARIs) during the COVID-19 pandemic and after the easing of COVID-19 restrictions. Our study investigated the epidemiology of 13 respiratory pathogens in Shijiazhuang, China, from January 2021 to December 2023. Thisese data isare crucial for the ongoing surveillance of epidemiological shifts in respiratory pathogens during and post the -COVID-19 pandemic, and serves as a scientific foundation for the prevention and management of ARIs.

## INTRODUCTION

Acute respiratory infections (ARIs) are a widespread disease leading to numerous emergency department visits and hospitalizations each year, marked by high morbidity and high transmissibility (1–4). Acute febrile respiratory illness is one of the main causes of hospitalization (5, 6). Pathogens responsible for ARIs encompass influenza viruses, respiratory syncytial viruses, rhinoviruses, human metapneumovirus, human coronaviruses, and human adenoviruses, among others (7–9). Variation in the prevalence of respiratory pathogens among countries and regions has been demonstrated in epidemiological studies, attributed to disparities in geographical location, humidity levels, and other influencing factors (10–12).

Coronavirus disease 2019 (COVID-19) pandemic, an outbreak caused by the severe acute respiratory syndrome coronavirus 2 (SARS-CoV-2) virus in December 2019, is a global public health disaster. The World Health Organization declared COVID-19 a public health emergency of international concern on 30 January 2020. A range of non-pharmaceutical interventions (NPIs) were implemented to curb the spread of SARS-CoV-2, including mask usage, proper hand hygiene, isolatingon of infected individuals, and vaccine administeringration vaccination. The successful implementation of stringent NPIs effectively reduced the transmission of COVID-19 and concurrently impacted the prevalence of other respiratory pathogens (13). The decreased exposure to common pathogens may result in the development of an immunity gap or immunity debt, making individuals more vulnerable to future infections by respiratory pathogens (14, 15). After the relaxation of NPIs in several countries, there have been documented instances of resurgence of respiratory pathogens in different geographical areas (16, 17). On 26 December 26, 2022, China terminated its dynamic zero-case policy, loosened COVID-19 restrictions, and implemented measures against Class-B infectious diseases (18). Subsequently, following the easing of NPIs, there was a notable increase in ARIs caused by various respiratory pathogens in China in 2023.

Previous studies have examined the impact of COVID-19-related NPIs, with a primary focus on the period of strict implementation (19, 20). However, the potential long-term consequences of the continued circulation of common respiratory pathogens following the relaxation of these interventions remain uncertain. This study specifically investigates the epidemiology of 13 respiratory pathogens both before and after the easing of NPIs, offering valuable insights for the prevention and management of ARIs in the post-COVID-19 era.

## MATERIALS AND METHODS

Nasopharyngeal swabs were collected from outpatients and inpatients presenting with acute respiratory infection symptoms (fever, cough, nasal obstruction, expectoration, sneezing, sore throat, dyspnea) or pneumonia diagnosed by chest radiography at the Hebei General Hospital from January 2021- to December 2023. Specimens were stored in sterile collection tubes at 2–8°C for no more than 8 hours until testing. The detection procedure comprises two steps: the initial step was total nucleic acid extraction using the Nucleic Acid Extraction or Purification Kit (Ning Bo Health Gene Technologies Co., Ltd, 1060167), and the second step was multiplex PCR and capillary electrophoresis to detect the 13 respiratory pathogens using the Respiratory Pathogen Multiplex Kit (Ning Bo Health Gene Technologies Co., Ltd, 1060071). The 13 respiratory pathogens included the Iinfluenza A virus (FluA: H7N9, H1N1, H3N2, H5N2): the; Iinfluenza A virus H1N1 (2009) (FluA-H1N1), the; Iinfluenza A virus H3N2 (FluA-H3N2); the Iinfluenza B virus (FluB: Victoria and Yamagata); the Pparainfluenza virus (PIV: I-–IV); the Aadenovirus (ADV: B, C, and E); the Rrhinovirus (RVs); the Mmetapneumovirus (MPV); theRrespiratory syncytial virus (RSV: A and B); the Ccoronavirus (CoV: 229E, OC43, NL63, and HKU1); the Mmycoplasma pneumonia (MP); theCchlamydia; and the Bbocavirus (BoV). We extracted patient demographic data (sampling time, clinical diagnosis, age, gender, and name) and laboratory test results from the Hebei General Hospital laboratory information system (LIS).

Patients were classified into six distinct age groups: Iinfants (< 1 year old), Ttoddlers (1–3 years old), Ppreschool children (3–6 years old), Sschool children (6–14 years old), Aadults (14–60 years old), and Eelders (>60 years old). The seasons are delineated as winter (December 1 to February 28), spring (March 1 to May 31), summer (June 1 to August 31), and autumn (September 1 to November 30). Given that our data collection ended on 31 December 31, 2023, only one month of winter was observed in 2023 (December 1 - –December 31).

The Hebei General Hospital, a comprehensive tertiary medical facility in Shijiazhuang, accommodatesd 2,206 open beds and recorded 1.39 million outpatient and emergency department visits, as well as 97,545 discharges, in 2023. Shijiazhuang, the capital of Hebei Province in Northern China, has a temperate continental monsoon climate with four distinct seasons.

### Statistical analysis

Categorical variables were expressed as numbers, percentages (%), and positiveity rates (%). The age distribution was not normal, so we calculated the median and interquartile range (IQR). This study used SPSS version 25.0 for data analysis and GraphPad Prism version 9.3.1 to visualize the data. Pearson’s Cchi-square or Fisher’s test wereas used to evaluate significance, and *P* values < 0.05 were considered statistically significant.

## RESULTS

### General characteristics of the study population

Table 1 presenteds the general characteristics of the study population. A total of 6,756 patients with ARIs were enrolled for sample analysis,; among them, 123 were excluded due to insufficient clinical data and/or repeated pathogen detection. Of the remaining 6,633 patients, 3,899 (58.78%) were male and 2,734 (41.22%) were female, with a male-to-female ratio of 1.42. No statistically significant difference in gender composition was observed annually (*P* > 0.05). The median age of the patient population iwas 58 years, with median ages of 16 years in 2021, 25 years in 2022, and 66 years in 2023. Among the patients, there were 173 patients under 1 year old (2.6%), 456 patients aged 1–3 years (6.87%), 724 patients aged 3–6 years (10.92%), 661 patients were over 6 years old (9.97%), and 1,408 patients aged 14–60 years (21.23%). The largest age group, comprising 3,211 patients (48.41%), was over 60 years old. Notably, the number of patients with ARIs seeking medical care at Hebei gGeneral hHospital increased significantly from 1,180 in 2021 and 1,161 in 2022 (COVID-19 pandemic phase) to 4,292 in 2023 (post-COVID-19 phase).

**TABLE 1:** Demographic data and positiveity rates of patients with ARIs^*a*^

Demographic		COVID-19				Post- COVID-19		Total	
		2021		2022		2023		2021––2023	
		Total	Positive n(%)	Total	Positive n(%)	Total	Positive n(%)	Total	Positive n(%)
Total		1,180	326(27.63)	1,161	283(24.38)	4,292	1,486(34.62*)	6,633	2,095(31.58)
Gender	Male	710	199(28.03)	667	157(23.54)	2,522	849(33.66*)	3,899	1,205(30.91)
	Female	470	127(27.02)	494	126(25.51)	1,770	637(35.99*)	2,734	890(32.55)
Age (year)		16[(3–66])	4[ (2–10.5])	25[(4–69])	5 [(3–18)	66(27–81)	33(6–72)	58(8–77)	11(4-67)
Age group	Infants (, <1)	52	16(30.77)	47	15(31.91)	74	46(62.16*)	173	77(44.51)
	Toddlers[1, 3)	186	77(41.4)	130	49(37.69)	140	81(57.86*)	456	207(45.39)
	Preschool children[3, 6)	229	110(48.03)	216	79(36.57)	279	190(68.1*)	724	379(52.35)
	Schoolchildren [6, 14)	115	45(39.13)	147	64(43.54)	399	318(79.7*)	661	427(64.6)
	Adults [14, 60)	232	33(14.22)	244	36(14.75)	932	285(30.58*)	1,408	354(25.14)
	Elders [60, )	366	45(12.3)	377	40(10.61)	2,468	566(22.93*)	3,211	651(20.27)

^*a*^ Values presented in this table are numbers and positivity rate (%). Age is represented by the median and interquartile range. The overall positivity rate, as well as the positivity rate for different genders and age groups, increased significantly in 2023 compared to 2021 and 2022 (*P < 0.05). Superscripts like “∗” are *P* values ; “∗” is a *P* value < 0.05.

### Overall positive detection

**Fig 1:**
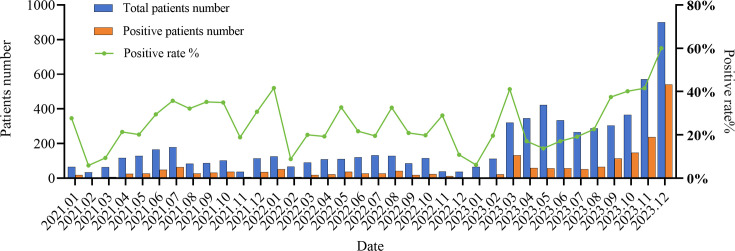
Overall number and positive number (positivity rate) of ARI patients in each month from 2021 to 2023

Figure 1 shows the total number and positive number (positivity rate) of ARI patients in each month from 2021 to 2023. We can see, except in January 2023, the number of ARI patients and positive patients in each month in 2023 was higher than the same period in 2021 and 2022. Table 1 illustrates at least one respiratory pathogen was detected in 2,095 (31.58%) patients, the positive detection rate of respiratory pathogens was 27.63% in 2021 and 24.38% in 2022. In 2023, the positivity rate increased to 34.62%, which was significantly higher than that in 2021 and 2022 (*P* < 0.05). From Table 2, we can see the top five dominant pathogens were FluA (7.57%), RV (7.1%), MP (6.42%), ADV (3.21%), and RSV (2.32%). The kit also enables the subtyping detection of FluA. The overall positive detection rates for FluA-H3N2 and FluA-H1N1 were 5.49% and 1.31%, respectively, indicating a significantly higher positivity rate for FluA-H3N2 compared to FluA-H1N1 (*P* < 0.05).

**TABLE 2:** Epidemiological characteristics of every respiratory pathogen detected in patients with ARIs^*a*^^,^^*b*^

Demographic		FluA	FluA- H1N1	FluA- H3N2	FluB	PIV	ADV	RV	MPV	RSV	CoV	MP	Chlamydia	BoV
Total		502(7.57)	87(1.31)	364(5.49)	70(1.06)	154(2.32)	213(3.21)	471(7.1)	163(2.46)	154(2.32)	70(1.06)	426(6.42)	13(0.20)	50(0.75)
Gender	Male	296(7.59)	58(1.49)	210(5.39)	43(1.1)	79(2.03)	124(3.18)	277(7.1)	102(2.62)	87(2.23)	48(1.23)	226(5.8)	10(0.26)	32(0.82)
	Female	206(7.53)	29(1.06)	154(5.63)	27(0.99)	75(2.74)	89(3.26)	194(7.1)	61(2.23)	67(2.45)	22(0.8)	200(7.32)	3(0.11)	18(0.66)
Age group	Infants (, <1)	3(1.73)	0(0.00)	2(1.16)	1(0.58)	10(5.78)	3(1.73)	12(6.94)	8(4.62)	32(18.5)	1(0.58)	3(1.73)	2(1.16)	6(3.47)
	Toddlers [1, 3)	8(1.75)	1(0.22)	4(0.88)	8(1.75)	26(5.7)	18(3.95)	77(16.89)	21(4.61)	34(7.46)	5(1.1)	14(3.07)	0(0)	21(4.61)
	Preschool children [3, 6)	40(5.52)	14(1.93)	19(2.62)	10(1.38)	46(6.35)	35(4.83)	110(15.19)	55(7.6)	45(6.22)	6(0.83)	59(8.18)	0(0)	8(1.1)
	Schoolchildren [6, 14)	29(4.39)	5(0.76)	18(2.72)	21(3.18)	10(1.51)	38(5.75)	69(10.44)	16(2.42)	3(0.45)	9(1.36)	273(41.3)	5(0.76)	0(0)
	Adults [14, 60)	119(8.45)	15(1.07)	87(6.18)	17(1.21)	12(0.85)	42(2.98)	68(4.83)	23(1.63)	13(0.92)	13(0.92)	67(4.76)	3(0.21)	9(0.64)
	Elders [60, )	303(9.44)	52(1.62)	234(7.29)	13(0.4)	50(1.56)	77(2.4)	135(4.2)	40(1.25)	27(0.84)	36(1.12)	10(0.31)	3(0.09)	6(0.19)
Year	2021	2(0.17)	2(0.17)	0(0.00)	17(1.44)	36(3.05)	22(1.86)	178(15.08)	22(1.86)	35(2.94)	4(0.34)	12(1.02)	0(0)	14(1.19)
	2022	12(1.03)	0(0.00)	10(0.86)	35(3.01)	32(2.76)	20(1.72)	56(4.82)	46(3.96)	8(0.69)	10(0.86)	75(6.46)	2(0.17)	5(0.43)
	2023	488(11.37)	85(1.98)	354(8.25)	18(0.42)	86(2.00)	171(3.98)	237(5.52)	95(2.21)	111(2.59)	56(1.30)	340(7.92)	10(0.23)	31(0.72)
Season														
2021	Spring (Mar–May)	0(0.00)	0(0.00)	0(0.00)	3(0.97)	4(1.29)	1(0.32)	40(12.90)	4(1.29)	6(1.94)	0(0.00)	0(0.00)	0(0.00)	0(0.00)
	Summer (June–Aug)	2(0.47)	2(0.47)	0(0.00)	1(0.23)	24(5.59)	11(2.56)	81(18.88)	5(1.17)	7(1.63)	0(0.00)	3(0.70)	0(0.00)	13(3.03)
	Autumn (Sept–Nov)	0(0.00)	0(0.00)	0(0.00)	1(0.44)	8(3.51)	6(2.63)	49(21.49)	2(0.88)	9(3.95)	1(0.44)	4(1.75)	0(0.00)	1(0.44)
	Winter (Dec–Feb)	0(0.00)	0(0.00)	0(0.00)	44(14.33)	0(0.00)	9(2.93)	11(3.58)	14(4.56)	9(2.93)	2(0.65)	10(3.26)	0(0.00)	0(0.00)
2022	Spring (Mar–May)	0(0.00)	0(0.00)	0(0.00)	3(0.97)	12(3.88)	5(1.62)	22(7.12)	19(6.15)	3(0.97)	1(0.32)	12(3.88)	1(0.32)	0(0.00)
	Summer (Jun–Aug)	6(1.57)	0(0.00)	4(1.05)	0(0.00)	19(4.97)	5(1.31)	17(4.45)	14(3.66)	2(0.52)	4(1.05)	33(8.64)	0(0.00)	2(0.52)
	Autumn (Sept–Nov)	7(2.92)	0(0.00)	6(2.5)	0(0.00)	1(0.42)	2(0.83)	10(4.17)	4(1.67)	0(0.00)	3(1.25)	24(10.00)	0(0.00)	2(0.83)
	Winter (Dec–Feb)	12(5.61)	9(4.21)	3(1.40)	0(0.00)	0(0.00)	3(1.40)	8(3.74)	2(0.93)	0(0.00)	0(0.00)	5(2.34)	3(1.40)	0(0.00)
2023	Spring (Mar–May)	130(11.95)	76(6.99)	50(4.60)	0(0.00)	0(0.00)	23(2.11)	44(4.04)	4(0.37)	43(3.95)	2(0.18)	2(.18)	0(0.00)	7(0.64)
	Summer (Jun–Aug)	3(0.34)	0(0.00)	2(0.23)	1(0.11)	38(4.28)	25(2.82)	30(3.38)	21(2.37)	9(1.01)	14(1.58)	37(4.17)	0(0.00)	8(0.90)
	Autumn (Sept–Nov)	71(5.73)	0(0.00)	70(5.65)	1(0.58)	40(3.23)	46(3.71)	117(9.44)	41(3.31)	12(0.97)	22(1.77)	187(15.08)	5(0.40)	9(0.73)
	Winter (Dec)	271(30.11)	0(0.00)	229(25.44)	16(1.78)	8(0.89)	76(8.44)	38(4.22)	28(3.11)	47(5.22)	18(2.00)	110(12.22)	4(0.33)	8(0.78)

^*a*^ Values presented in this table are numbers and positivity rate (%).

^*b*^ FluA, influenza A virus; FluA-H1N, influenza A virus H1N1; FluA-H3N2, influenza A virus H3N2; FluB, influenza B; PIV, parainfluenza virus; ADV, adenovirus; RV, rhinovirus; MPV, metapneumovirus; RSV, respiratory syncytial virus; CoV, coronavirus; MP, mycoplasma pneumonia; BoV, bocavirus.

### Co-infectious pattern

**Fig 2:**
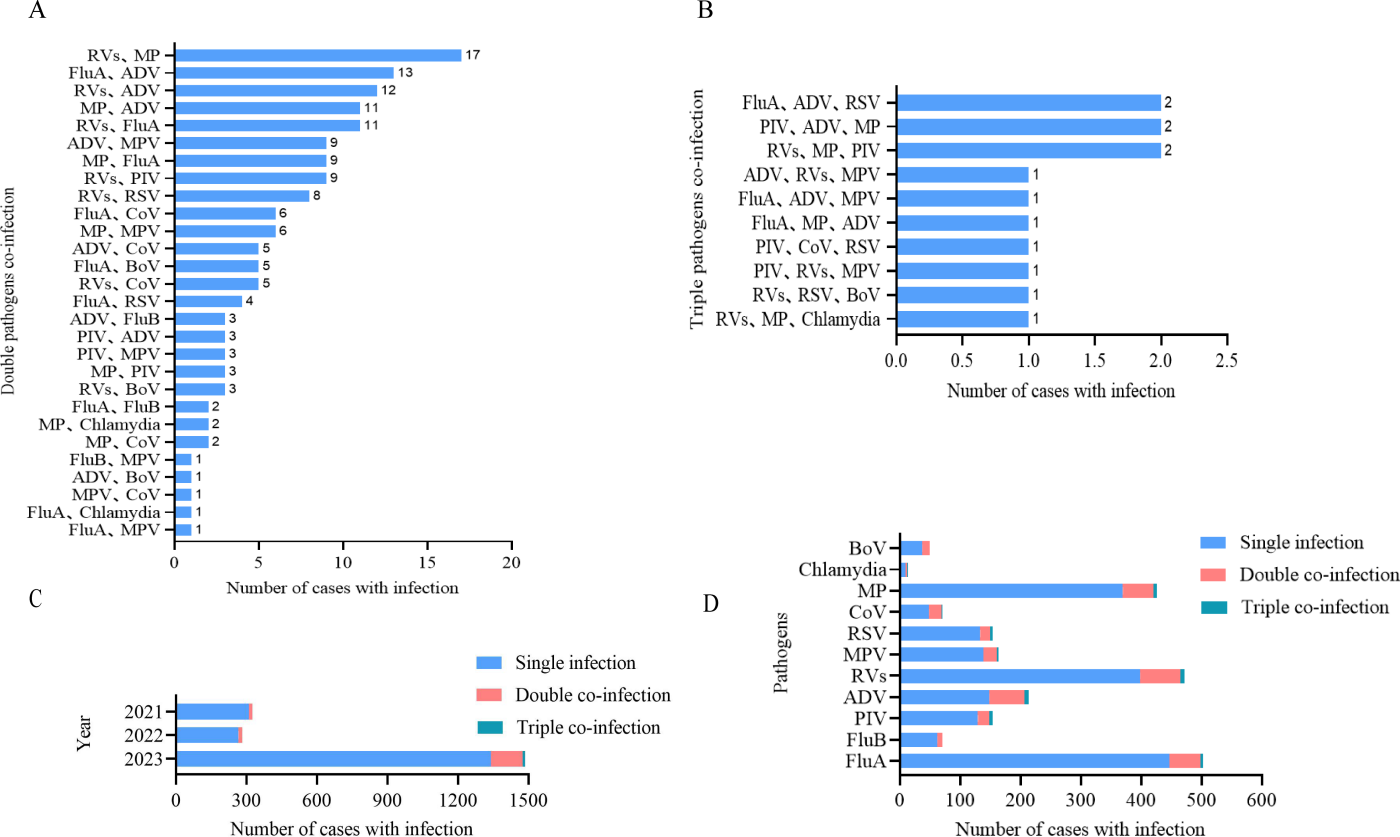
The co-infection pattern of respiratory pathogens detected in patients with ARIs. (**A**) Double pathogen co-infection. (**B**) Triple pathogen co-infection. (**C**) Infection patterns in different years. (**D**) Infection patterns of every respiratory pathogen. FluA, influenza A; FluB, influenza B; PIV, parainfluenza virus; ADV, adenovirus; RV, rhinovirus; MPV, metapneumovirus; RSV, respiratory syncytial virus; CoV, coronavirus; MP, mycoplasma pneumonia; BoV, bocavirus.

Table S1 illustrates the co-infectious pattern. Due to multiple co-infections, the number of infected pathogens was higher than the number of infected individuals. A total of 2,286 positive pathogens were detected in 2,095 patients. Among these patients, 1,917 were found to be infected with a single pathogen, 165 with a co-infection of two pathogens, and 13 with a co-infection of three pathogens. RV was the most common co-infecting pathogen, with 67 cases of double infection and six cases of triple infection. The most prevalent double-infection pattern observed was RV + MP, which was present in 17 patients. The patterns of double infection and triple infection are illustrated in Fig. 2A and B. From Fig. 2C, we can see the percentage of co-infections occurring in 2023 was significantly higher than that in 2021 and 2022 (*P* < 0.05). These co-infections occurred more frequently after easing restrictions. Figure 2D displays the infection patterns of various respiratory pathogens in patients with ARIs.

### Gender distribution of ARI patients positive for respiratory pathogens

From Table 1, we can see there were 1,205 males and 890 females detected positive for respiratory pathogens, with a male-to-female ratio of 1.35. There was no significant difference in gender composition and in positive detection rates between males and females annually (*P* > 0.05). In the year 2023, the total positive detection rate for males was higher compared to the years 2021 and 2022, as was also observed for females (*P <* 0.05). As shown in Table 2, there was no significant difference in the positive detection rates for each pathogen between males and females (*P* > 0.05), except for MP (*P* < 0.05). The positive detection rate of MP in females (7.32%) was significantly higher than in males (5.8%).

### Age distribution of ARI patients positive for respiratory pathogens

**Fig 3:**
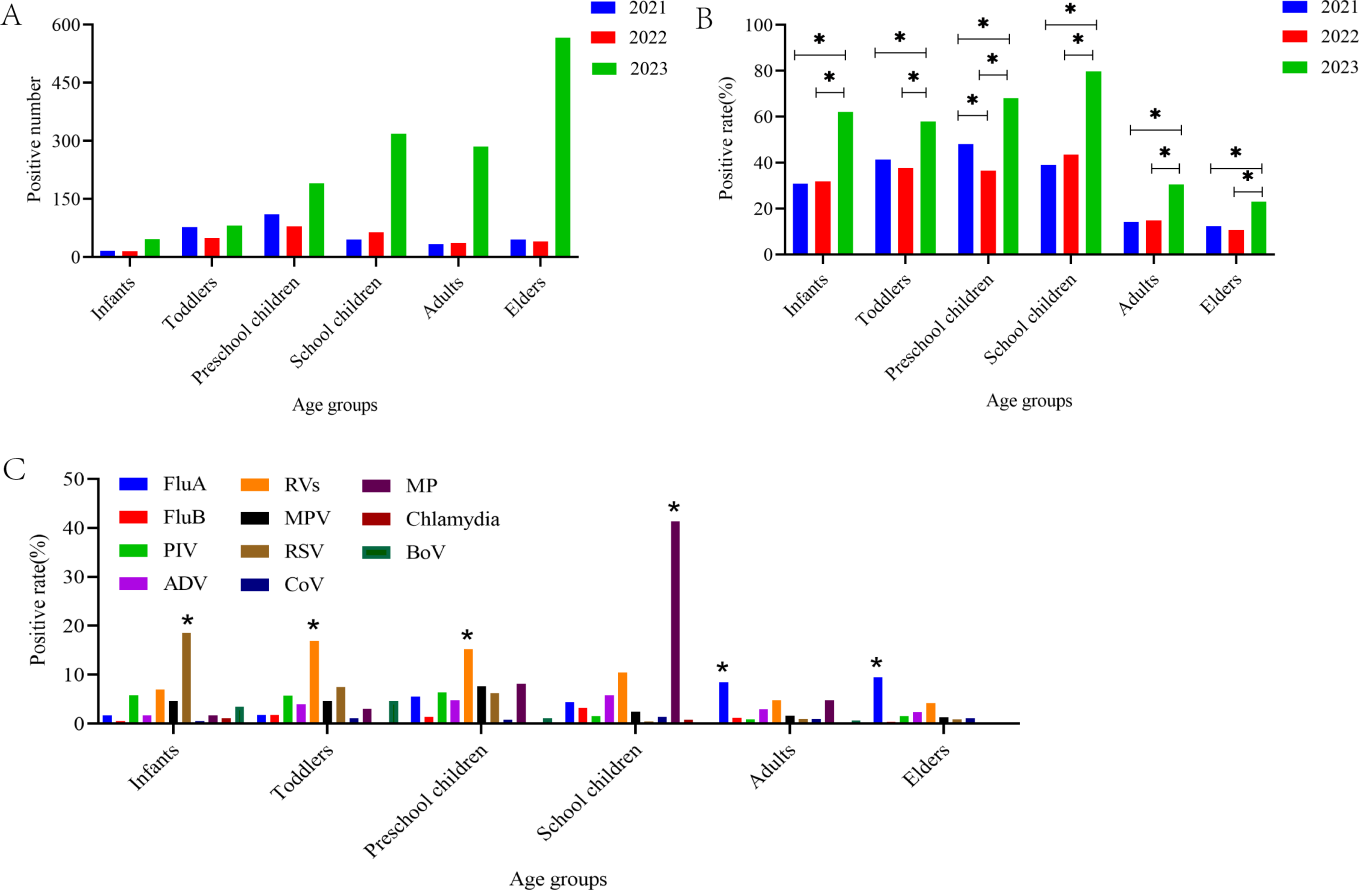
Age distribution of ARI patients who detected positive for various respiratory pathogens. (**A**) Positive number of ARIs among various age groups. The positive number among various age groups in 2023 was higher compared to 2021 and 2022. (**B**) Positivity rate of ARIs among various age groups. The positivity rates among various age groups in 2023 were higher compared to 2021 and 2022 (*P < 0.05). (**C**) Age distribution of every respiratory pathogen. Infants exhibited greater susceptibility to RSV (*P < 0.05). Toddlers and preschool children were more likely to contract RV (*P < 0.05). Schoolchildren were more commonly infected by MP (*P < 0.05). Adults and elders were more prone to FluA (*P < 0.05). Superscripts like “∗” are *P* values; “∗” is a *P* value < 0.05.

From Table 1, we can see the median age of the positive patients was 11 years old. The annual median age was 4 in 2021, 5 in 2022, and 33 in 2023. The higher positivity rate of infection over the 3-year period was observed in patients under the age of 14. Schoolchildren (64.6%) had the highest positivity rate of pathogen detection, followed by preschool children (52.35%), toddlers (45.39%), infants (44.51%), adults (25.14%), and elders (20.27%). Figure 3 illustrates a significant increase in the positive number and positivity rates among various age groups in 2023 compared to 2021 and 2022 (*P* < 0.05). In Fig. 3C, the age distribution of various respiratory pathogens indicates that infants exhibited a higher susceptibility to RSV (18.5%) (*P* < 0.05), whereas toddlers and preschoolers showed higher positivity rates of RV (16.89% and 15.19%, respectively) (*P* < 0.05). MP predominantly affected school-aged children between 6 and 14 years old (*P* < 0.05), with a notably high positivity rate of 41.3% in this demographic. Adults and the elderly were more prone to infection with FluA, with positivity rates of 8.45% and 9.44%, respectively (*P* < 0.05).

### Changes of epidemiological trends of respiratory pathogens from 2021 to 2023

**Fig 4:**
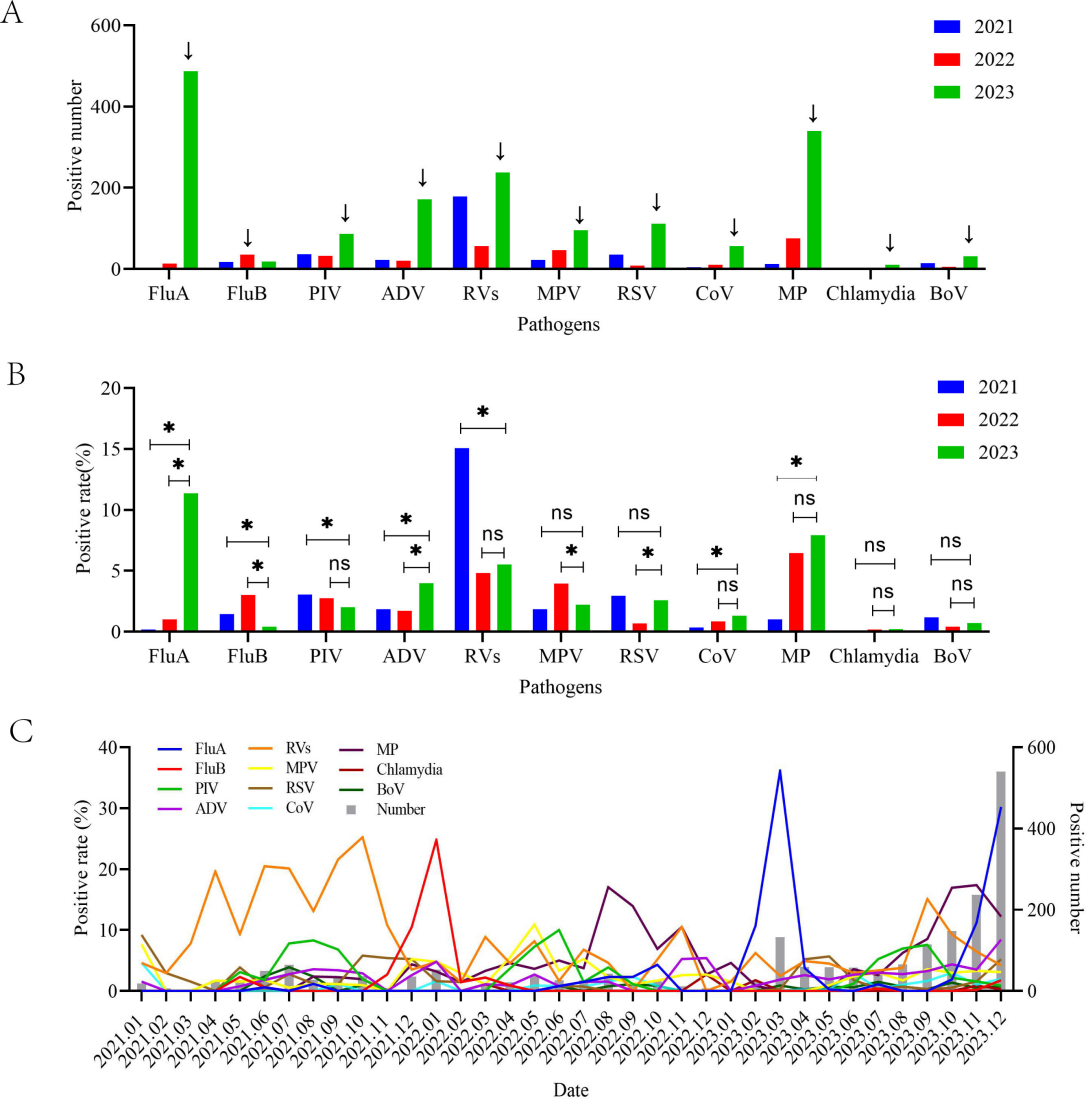
Prevalence of every respiratory pathogens during the COVID‐19 pandemic and after the easing of COVID‐19 restrictions. (**A**) Positive number of every respiratory pathogen in 2021, 2022, and 2023. (**B**) Positivity rate of every respiratory pathogen in 2021, 2022, and 2023.(**C**) Changes of prevalence trends of every respiratory pathogen spanning from January 2019 to December 2023. Colored lines represent the positivity rate of every respiratory pathogen over time, and gray bars represent the positive patient number over time. Superscripts like “∗” are *P* values ; “∗” is a *P* value < 0.05; “ns“ is a *P* value > 0.05. Superscripts like “↓” indicate the maximum value of the histograms in 2021, 2022, and 2023.

As illustrated in Table 2 and Fig. 4, we can see, following the relaxation of NPIs in 2023, the infection numbers of various respiratory pathogens exhibited a resurgence, with the exception of FluB. However, the alterations in positivity rates differed among the pathogens.

#### FluA and ADV

In 2023, there was a simultaneous rise in the number of positive cases and positivity rates for both FluA and ADV, as compared to the preceding years of 2021 and 2022. FluA detected only two positive cases (0.17%) in 2021 and 13 (1.02%) in 2022. However, 487 FluA positive cases (11.35%) were detected in 2023, accounting for 32.77% of the total number of positive cases in 2023, which increased sharply compared to 2021 and 2022 (*P* < 0.05), and peaked in spring (11.95%) and winter (30.11%). Similarly, ADV was detected in 171 patients in 2023, with a positivity rate of 3.98%, which is significantly higher than 22 (1.86%) in 2021 and 20 (1.72%) in 2022 (*P* < 0.05).

#### RSV, MP, and CoV

In 2023, the rates of infection for certain pathogens, including RSV, MP, and CoV, were either higher or equivalent to those observed during the COVID-19 pandemic. Specifically, cases of RSV surged to 111 in 2023, a marked increase compared to the preceding years of 2021 and 2022. The positivity rate in 2023 (2.59%) was also higher than that in 2022 (0.69%) (*P* < 0.05) but not statistically different from that in 2021 (2.94%) (*P* > 0.05). Interestingly, RSV deviated from its typical seasonal epidemic trend in 2021 and 2022, and reverted back to this pattern in 2023, peaking in spring and winter. In 2022, MP emerged as the predominant respiratory pathogen, infecting 75 individuals and representing 26.5% of all reported cases. MP infections surged to 340 in 2023, with a positivity rate of 7.92%, peaking in autumn (15.08%) and winter (12.22%). Notably, schoolchildren aged 6–14 years were particularly affected, with a positivity rate of 41.3% within this demographic. Despite the substantial increase in positive cases in 2023, the positivity rate did not exhibit a statistically significant difference compared to 2022 (6.46%) (*P* > 0.05) but was notably higher than that observed in 2021 (1.02%) (*P* < 0.05). The same trend was observed for CoV. The positivity of CoV in 2023 (1.30%) was higher than that in 2021 (0.34%) (*P* < 0.05) but not significantly different from that in 2022 (0.86%) (*P* > 0.05).

#### BoV and chlamydia

The incidence of BoV and chlamydia infections remained low over the preceding 3 years, with a slight increase in positive individuals observed in 2023; however, no significant disparities were observed in the positivity rates of infections (*P* > 0.05).

#### PIV, RV, and MPV

The infection positivity rates of certain respiratory pathogens, such as PIV, RV, and MPV, were observed to be lower in 2023 compared to the period of the COVID-19 pandemic. Specifically, the positivity rates of PIV and RV in 2023 were significantly lower than those in 2021 (*P* < 0.05). Additionally, the seasonal epidemic pattern of PIV was also not affected from 2021 to 2023, with annual peaks occurring in the summer months and with positivity rates of 5.59%, 4.97%, and 4.28%, respectively. Although the prevalence of influenza and other respiratory viruses was greatly affected by the NPIs, there was still a concentrated outbreak of RV infections in 2021, peaking in autumn (21.49%), with 178 infected individuals, accounting for 54.6% of all positive pathogens in that year. The positivity rate of RV infections was as high as 15.08% in 2021, which is significantly higher than that in 2022 (4.82%) and 2023 (5.52%) (*P* < 0.05). In 2021–2023, MPV infections numbered 22 (1.86%), 46 (3.96%), and 95 (2.21%), peaking in winter 2021 (4.56%) and spring 2022 (6.15%). The positivity rate for MPV infections in 2023 was lower than that in 2022 (*P* < 0.05) and not significantly different from that in 2021 (*P* > 0.05).

#### FluB

The positivity rate of FluB in 2023 was 0.42%, a statistically significant decrease from the rates of 1.44% in 2021 and 3.01% in 2022 (*P* < 0.05). Additionally, the number of positive FluB cases in 2023 was lower compared to 2022.

## DISCUSSION

The COVID-19 pandemic, caused by SARS-CoV-2, has disrupted daily life globally, resulting in unprecedented numbers of cases and deaths. NPIs (global travel cessation, mask wearing, social distancing, hand hygiene, and home isolation) were implemented almost simultaneously worldwide, aiming to slow community SARS-CoV-2 transmission, reduce disease burden and medical resource demand (21). During the COVID-19 outbreak, China initially implemented containment measures, followed by adoption of a dynamic zero-case policy. NPIs were used continuously until 26 December 2022 when the COVID-19 management level was reduced to Class B (18) and social activities gradually resumed.

Our retrospective study investigated the epidemiological characteristics of 13 respiratory pathogens during the COVID-19 pandemic (January 2021–December 2022) and after easing COVID-19 restrictions (January 2023–December 2023) in Shijiazhuang, China. In our study, at least one respiratory pathogen was detected in 2,095 (31.58%) of 6,633 patients, generally consistent with surveillance in other countries (11, 22). The number of positive patients with ARIs and the pathogen detection rate were kept at a lower level from 2021 (326, 27.36%) to 2022 (283, 24.38%) (during the COVID-19 pandemic) but saw a large increase in 2023 (1,486, 34.62%) (after the easing of COVID‐19 restrictions). The phenomenon suggests that NPIs implemented nationwide to curb COVID-19 were equally effective in reducing other respiratory pathogen transmission (23). Furthermore, the significant increase in ARIs in 2023 was linked to a resurgence of respiratory pathogen infections subsequent to the relaxation of NPIs (24, 25). After the relaxation of NPIs in 2023, the positive cases and positivity rate of respiratory pathogens increased among all age groups (Fig. 3). Children under 14 years of age showed a greater increase in positivity rates than that in adults and the elderly. The reason may be because schools and kindergartens postponed or suspended classes to avoid gatherings during the COVID-19 pandemic, but when the COVID-19 restrictions eased, children under 14 gathered in schools and kindergartens, which made respiratory pathogens to be transmitted easier (26, 27), spurring a larger rise in infection positivity rate. In 2023, adults and the elderly were affected by an outbreak of FluA infections, with a surge in the number of positive infections. However, the increase in positivity rates was lower than in people under 14 years of age, possibly because after 2023, more adults and older adults sought medical attention at the Hebei General Hospital and were more willing to undergo multiple nucleic acid testing for respiratory pathogens.

Figure 1 shows that after restrictions were lifted on 26 December 2022, the total number of patients positive for ARIs increased. However, the positivity rate did not appear to differ significantly from before, fluctuating up and down between 2021 and 2023, and reaching a peak of 60% in December 2023. This phenomenon may be caused by the following reasons: during 2021–2022, China implemented NPIs under a dynamic zero-case policy. Communities reporting COVID-19 cases were locked down, limiting population mobility and respiratory pathogen transmission. However, in unsealed areas, uninterrupted social interactions led to ongoing spread of respiratory pathogens. Simultaneously, people’s willingness to seek medical care diminished, with only patients presenting severe symptoms of ARIs willing to visit hospitals, resulting in an increased positive detection rate. After 26 December 2022, increased mobility and heightened inclination among individuals with ARIs to seek hospital care expanded the tested pool, which may have resulted in relatively lower positivity rates. In December 2023, large-scale explosive infections of FluA and MP directly led to a significant increase in positive cases, making the positivity rate of infection reached a peak of 60%.

After the easing of restrictive NPIs, the number of positive cases for each respiratory pathogen increased except for FluB (Fig. 4A), yet from Fig. 4B, we can see the positivity rates did not necessarily increase simultaneously. Our study found that FluA was most greatly affected by the NPIs, with the most obvious inhibitory effects in 2021–2022 and the rebound effects in 2023. Influenza was the most common pathogen in patients with ARIs, as reported before. With the implementation of NPIs in the early stages of COVID-19, global influenza virus activity decreased significantly (28–30). Feng et al. (31) found that during the COVID-19 pandemic, COVID-19-related NPIs reduced influenza in Northern China by 79.4% (44.9%–87.4%). Our study showed a significant decrease in FluA infections during the COVID-19 pandemic in 2021–2022, with no seasonal pattern. In 2023, the infections increased significantly after the easing of COVID‐19 restrictions and resumed a seasonal pattern similar to previous surveys in northern China (32). The NPIs also have a large impact on other respiratory pathogens. We found that the number of positive cases of MP surged to 340 in 2023, higher than 12 in 2021 and 75 in 2022. Other countries and regions have also seen a delayed but significant rebound of MP infections after the relaxation of COVID-19 control measures (25, 33, 34). In our study, RSV cases rebounded to 111 in 2023, significantly higher than 35 in 2021 and 8 in 2022 (*P* < 0.05). The number of RSV-positive patients and hospitalizations increased after easing strict NPIs. This may be due to a declining herd immunity during the COVID-19 pandemic (16, 35–37). However, the positivity rate of RSV in 2023 was not significantly different from that in 2021. Xu et al. (38) also observed the same phenomenon that there was no significant difference in RSV positivity rate between the pre-COVID-19 pandemic and pandemic periods (5.6% vs 5.8%, *P* = 0.117). We also found that the seasonal epidemic pattern of PIV was not affected during the COVID-19 pandemic. Additionally, in 2021, the positivity rate of PIV was significantly higher compared to 2023 (*P* < 0.05). Kim et al. (39) also reported a similar phenomenon: the Republic of Korea experienced a delayed and unprecedentedly high detection of PIV during the COVID-19 pandemic, according to both sentinel and non-sentinel surveillance. Li et al. (40) found the detection rates of PIV increased in Southern China during the COVID-19 outbreak, which underlines the importance of a continuous monitoring of PIV in the next epidemic season. Also noteworthy is RV, which saw a spike in infections in 2021 under strict NPI policies during the COVID-19 pandemic. We found that RV was the dominant pathogen in 2021, with a positivity rate of 15.08%, which was significantly higher than that in 2022 and 2023. Other studies also found that RV was a common pathogen in patients with ARIs during the COVID-19 pandemic in Xiamen (41) and the Philippines (42). In Canada, strict public health measures to control COVID-19 virtually suppressed most seasonal respiratory viruses, except for RV/Enterovirus (EV). RV can serve as a sentinel for population exposure levels to assess the effectiveness and necessity of public health measures and to predict future prevalence of other seasonal or emerging respiratory pathogens (43). It's worth noting that FluB showed a small peak of infection in 2022. Both the number of infections and the positivity rate of infection were higher than after the relaxation of NPI policy in 2023.

NPIs are designed to mitigate respiratory pathogen transmission. They significantly disrupt seasonal circulation patterns of common respiratory pathogens (including influenza, ADV, MP, and more). Emergency department visits and hospitalizations associated with non-SARS-CoV-2 respiratory viral infections decreased early in the COVID-19 pandemic (12). As reported in many countries, the prevalence of influenza and respiratory viruses was below normal, likely due to the NPIs (11). In the early stages of the COVID-19 pandemic, strict NPIs left people lacking immunity due to reduced circulation of respiratory pathogens, resulting in more susceptible people who could be infected and transmit respiratory pathogens after the relaxation of COVID-19 restrictions. Moreover, interactions between viruses can impact the occurrence of respiratory pathogen infections (44, 45). Additionally, infection with SARS-CoV-2 can compromise the immune system, reducing the body’s ability to resist other pathogens (46). These factors contributed to a notable resurgence of respiratory infections in China following the relaxation of NPIs in 2023.

However, the seasonal patterns of some respiratory pathogens, such as PIV, were barely affected by the NPI policy during the COVID-19 pandemic, and some respiratory viruses still showed outbreaks of infection, such as RV, which should take into account changes in population mobility and concentration. China’s zero-COVID strategy, including localized lockdowns in communities reporting COVID-19 cases, which limits population mobility and reduces the spread of respiratory pathogens, consequently reduced the cases of ARIs. However, in areas free of restrictions, uninterrupted social interactions lead to the ongoing spread of respiratory pathogens, especially seasonally circulating respiratory pathogens. In some cases, despite an overall decline in infections, clusters of activity in a particular region or population and the seasonal prevalence of respiratory pathogens can lead to an increase in local infection rates, ultimately impacting the overall positivity rate of detections conducted in those areas or populations. Furthermore, vaccination status is also an important factor affecting infection numbers and positivity rates. A comprehensive understanding of this phenomenon requires consideration of various factors such as prevention and control measures, detection technology, population mobility, and vaccination coverage.

Our research also has some limitations. Firstly, our data set was limited to a single hospital, potentially impacting the generalizability of the findings. Factors such as access to healthcare, severity of illness, and willingness to take the test could influence the sample composition. To enhance the credibility of results, future multicenter trials with larger cohorts will provide better reference and support for the prevention and control of ARIs. Secondly, our detection strategy utilized the Respiratory Pathogen Multiplex Kit (Ning Bo Health Gene Technologies Co., Ltd, 1060071), employing universal primers (47, 48) characteristic of enteroviruses. Adopting this inclusive methodology to detect the pathogen spectrum from rhinovirus A (RV-A) to RV-C inevitably encompasses other enteroviruses. Due to the inherent limitations of our methodology, it is not possible to fully discriminate between RV and other enteroviruses at the technical level. Ongoing research efforts will be dedicated to refining this aspect. Thirdly, the current testing panels for multiplex PCR may not cover all possible co-infecting viral, bacterial, or fungal respiratory pathogens. Subsequent studies should broaden the detection spectrum of respiratory pathogens, thereby enriching the epidemiological profile of ARIs. This underscores the imperative for a more comprehensive approach to respiratory infection surveillance in forthcoming research.

### Conclusion

The COVID-19 pandemic has caused significant fluctuations in the prevalence of non-SARS-CoV-2 respiratory pathogens in patients with ARIs. These shifts underscore the complex relationship between respiratory pathogen transmission, public health measures, and disease epidemiology. A continuous monitoring of the prevalence of respiratory pathogens is essential to inform targeted intervention strategies and vaccination programs, thereby facilitating the effective management of acute respiratory infections in the post-COVID-19 era.

## SUPPLEMENTAL MATERIAL

Tables S1 and S2 spectrum.01161-24-s0001.docx Table S1: Co-infection detected in patients with ARIs. Table S2: Total number and positive number (positivity rate) of ARI patients.

ASM does not own the copyrights to Supplemental Material that may be linked to, or accessed through, an article. The authors have granted ASM a non-exclusive, world-wide license to publish the Supplemental Material files. Please contact the corresponding author directly for reuse.

## References

[1] Moesker FM, van Kampen JJA, van Rossum AMC, de Hoog M, Koopmans MPG, Osterhaus A, Fraaij PLA. 2016. Viruses as sole causative agents of severe acute respiratory tract infections in children. PLoS ONE 11:e0150776. doi:10.1371/journal.pone.0150776 26964038 PMC4786225

[2] Cillóniz C, Pericàs JM, Rojas JR, Torres A. 2022. Severe infections due to respiratory viruses. SEMIN Respir CRIT CARE Med 43:60–74. doi:10.1055/s-0041-1740982 35172359

[3] Jones AH, Ampofo W, Akuffo R, Doman B, Duplessis C, Amankwa JA, Sarpong C, Sagoe K, Agbenohevi P, Puplampu N, Armah G, Koram KA, Nyarko EO, Bel-Nono S, Dueger EL. 2016. Sentinel surveillance for influenza among severe acute respiratory infection and acute febrile illness inpatients at three hospitals in Ghana. INFLUENZA Other Respir Viruses 10:367–374. doi:10.1111/irv.12397 27239956 PMC4947945

[4] Liu BM, Beck EM, Fisher MA. 2021. The brief case: ventilator-associated Corynebacterium accolens pneumonia in a patient with respiratory failure due to COVID-19. J Clin Microbiol 59:e0013721. doi:10.1128/JCM.00137-21 34406882 PMC8372998

[5] Gasem MH, Kosasih H, Tjitra E, Alisjahbana B, Karyana M, Lokida D, Neal A, Liang CJ, Aman AT, Arif M, Sudarmono P, Merati TP, Lisdawati V, Siddiqui S, Lane HC, INA-RESPOND. 2022. Correction: an observational prospective cohort study of the epidemiology of hospitalized patients with acute febrile illness in Indonesia. PLoS Negl Trop Dis 16:e0010530. doi:10.1371/journal.pntd.0010530 35679250 PMC9182548

[6] De Conto F, Conversano F, Medici MC, Ferraglia F, Pinardi F, Arcangeletti MC, Chezzi C, Calderaro A. 2019. Epidemiology of human respiratory viruses in children with acute respiratory tract infection in a 3-year hospital-based survey in Northern Italy. DIAGN Microbiol Infect DIS 94:260–267. doi:10.1016/j.diagmicrobio.2019.01.008 30745224 PMC7126416

[7] GBD 2019 Diseases and Injuries Collaborators. 2020. Global burden of 369 diseases and injuries in 204 countries and territories, 1990-2019: a systematic analysis for the Global Burden of Disease Study 2019. LANCET 396:1204–1222. doi:10.1016/S0140-6736(20)30925-9 33069326 PMC7567026

[8] Lauinger IL, Bible JM, Halligan EP, Bangalore H, Tosas O, Aarons EJ, MacMahon E, Tong CYW. 2013. Patient characteristics and severity of human rhinovirus infections in children. J CLIN VIROL 58:216–220. doi:10.1016/j.jcv.2013.06.042 23886500 PMC7108361

[9] Amer HM, Alshaman MS, Farrag MA, Hamad ME, Alsaadi MM, Almajhdi FN. 2016. Epidemiology of 11 respiratory RNA viruses in a cohort of hospitalized children in Riyadh, Saudi Arabia. J MED VIROL 88:1086–1091. doi:10.1002/jmv.24435 26595650 PMC7167021

[10] Dallmeyer LK, Schüz ML, Fragkou PC, Omony J, Krumbein H, Dimopoulou D, Dimopoulou K, Skevaki C. 2024. Epidemiology of respiratory viruses among children during the SARS-CoV-2 pandemic: a systematic review and meta-analysis. Int J Infect Dis 138:10–18. doi:10.1016/j.ijid.2023.10.023 37951460

[11] Li Z-J, Zhang H-Y, Ren L-L, Lu Q-B, Ren X, Zhang C-H, Wang Y-F, Lin S-H, Zhang X-A, Li J, et al.. 2021. Etiological and epidemiological features of acute respiratory infections in China. Nat Commun 12:5026. doi:10.1038/s41467-021-25120-6 34408158 PMC8373954

[12] Chow EJ, Uyeki TM, Chu HY. 2023. The effects of the COVID-19 pandemic on community respiratory virus activity. NAT REV MICROBIOL 21:195–210. doi:10.1038/s41579-022-00807-9 36253478 PMC9574826

[13] Oh D-Y, Buda S, Biere B, Reiche J, Schlosser F, Duwe S, Wedde M, von Kleist M, Mielke M, Wolff T, Dürrwald R. 2021. Trends in respiratory virus circulation following COVID-19-targeted nonpharmaceutical interventions in Germany, January - September 2020: analysis of national surveillance data. Lancet Reg Health Eur 6:100112. doi:10.1016/j.lanepe.2021.100112 34124707 PMC8183189

[14] Cohen R, Ashman M, Taha M-K, Varon E, Angoulvant F, Levy C, Rybak A, Ouldali N, Guiso N, Grimprel E. 2021. Pediatric Infectious Disease Group (GPIP) position paper on the immune debt of the COVID-19 pandemic in childhood, how can we fill the immunity gap? Infect Dis Now 51:418–423. doi:10.1016/j.idnow.2021.05.004 33991720 PMC8114587

[15] Baker RE, Park SW, Yang W, Vecchi GA, Metcalf CJE, Grenfell BT. 2020. The impact of COVID-19 nonpharmaceutical interventions on the future dynamics of endemic infections. Proc Natl Acad Sci U S A 117:30547–30553. doi:10.1073/pnas.2013182117 33168723 PMC7720203

[16] Eden J-S, Sikazwe C, Xie R, Deng Y-M, Sullivan SG, Michie A, Levy A, Cutmore E, Blyth CC, Britton PN, et al.. 2022. Off-season RSV epidemics in Australia after easing of COVID-19 restrictions. Nat Commun 13:2884. doi:10.1038/s41467-022-30485-3 35610217 PMC9130497

[17] Messacar K, Baker RE, Park SW, Nguyen-Tran H, Cataldi JR, Grenfell B. 2022. Preparing for uncertainty: endemic paediatric viral illnesses after COVID-19 pandemic disruption. LANCET 400:1663–1665. doi:10.1016/S0140-6736(22)01277-6 35843260 PMC9282759

[18] Notice on the issuance of the overall plan for the implementation of the "Class B " for the novel coronavirus infection. The Joint Prevention and Control Mechanism of The State Council for novel coronavirus infection. 2022. Available from: http://www.nhc.gov.cn/xcs/zhengcwj/202212/e97e4c449d7a475794624b8ea12123c6.shtml

[19] Sakamoto H, Ishikane M, Ueda P. 2020. Seasonal influenza activity during the SARS-CoV-2 outbreak in Japan. JAMA 323:1969–1971. doi:10.1001/jama.2020.6173 32275293 PMC7149351

[20] Li ZJ, Yu LJ, Zhang HY, Shan CX, Lu QB, Zhang XA, Ren X, Zhang CH, Wang YF, Lin SH, Xu Q, Jiang BG, Jiang T, Lv CL, Chen JJ, Gao GF, Yang WZ, Wang LP, Yang Y, Fang LQ, Liu W, Chinese Centers for Disease Control and Prevention (CDC) Etiology Surveillance Study Team of Acute Respiratory Infections. 2022. Broad impacts of coronavirus disease 2019 (COVID-19) pandemic on acute respiratory infections in China: an observational study. CLIN INFECT DIS 75:e1054–e1062. doi:10.1093/cid/ciab942 34788811 PMC8767888

[21] Liu P, Xu M, Cao L, Su L, Lu L, Dong N, Jia R, Zhu X, Xu J. 2021. Impact of COVID-19 pandemic on the prevalence of respiratory viruses in children with lower respiratory tract infections in China. Virol J 18:159. doi:10.1186/s12985-021-01627-8 34344406 PMC8329611

[22] Huang X-B, Yuan L, Ye C-X, Zhu X, Lin C-J, Zhang D-M, He K-S, Niu R-X, Cao K-Y, Xu L. 2020. Epidemiological characteristics of respiratory viruses in patients with acute respiratory infections during 2009-2018 in southern China. INT J INFECT DIS 98:21–32. doi:10.1016/j.ijid.2020.06.051 32562851

[23] Du X, Wu G, Zhu Y, Zhang S. 2021. Exploring the epidemiological changes of common respiratory viruses since the COVID-19 pandemic: a hospital study in Hangzhou, China. ARCH VIROL 166:3085–3092. doi:10.1007/s00705-021-05214-8 34480636 PMC8417671

[24] Looi MK. 2023. China: rising cases of respiratory disease and pneumonia spark WHO concern. BMJ:2770. doi:10.1136/bmj.p2770 37996101

[25] Parums DV. 2023. Editorial: outbreaks of post-pandemic childhood pneumonia and the re-emergence of endemic respiratory infections. Med Sci Monit 29:e943312. doi:10.12659/MSM.943312 38037346 PMC10702145

[26] Ye Q, Wang D. 2022. Epidemiological changes of common respiratory viruses in children during the COVID-19 pandemic. J MED VIROL 94:1990–1997. doi:10.1002/jmv.27570 34981839 PMC9015628

[27] Ye Q, Liu H. 2022. Impact of non-pharmaceutical interventions during the COVID-19 pandemic on common childhood respiratory viruses - An epidemiological study based on hospital data. MICROBES INFECT 24:104911. doi:10.1016/j.micinf.2021.104911 34871774 PMC8641407

[28] Laurie KL, Rockman S. 2021. Which influenza viruses will emerge following the SARS-CoV-2 pandemic? Influenza Other Respir Viruses 15:573–576. doi:10.1111/irv.12866 33955176 PMC8242426

[29] Olsen SJ, Azziz-Baumgartner E, Budd AP, Brammer L, Sullivan S, Pineda RF, Cohen C, Fry AM. 2020. Decreased influenza activity during the COVID-19 pandemic-United States, Australia, Chile, and South Africa, 2020. AM J TRANSPLANT 20:3681–3685. doi:10.1111/ajt.16381 33264506 PMC7753605

[30] Takeuchi H, Kawashima R. 2023. Disappearance and re-emergence of influenza during the COVID-19 pandemic: association with infection control measures. Viruses 15:223. doi:10.3390/v15010223 36680263 PMC9862942

[31] Feng L, Zhang T, Wang Q, Xie Y, Peng Z, Zheng J, Qin Y, Zhang M, Lai S, Wang D, Feng Z, Li Z, Gao GF. 2021. Impact of COVID-19 outbreaks and interventions on influenza in China and the United States. Nat Commun 12:3249. doi:10.1038/s41467-021-23440-1 34059675 PMC8167168

[32] Liao Y, Xue S, Xie Y, Zhang Y, Wang D, Zhao T, Du W, Chen T, Miao H, Qin Y, Zheng J, Yang X, Peng Z, Yu J. 2022. Characterization of influenza seasonality in China, 2010-2018: implications for seasonal influenza vaccination timing. INFLUENZA Other Respir Viruses 16:1161–1171. doi:10.1111/irv.13047 36062624 PMC9530570

[33] Meyer Sauteur PM, Beeton ML, European Society of Clinical Microbiology and Infectious Diseases (ESCMID) Study Group for Mycoplasma and Chlamydia Infections (ESGMAC), and the ESGMAC Mycoplasma pneumoniae Surveillance (MAPS) study group. 2024. Mycoplasma pneumoniae: delayed re-emergence after COVID-19 pandemic restrictions. Lancet Microbe 5:e100–e101. doi:10.1016/S2666-5247(23)00344-0 38008103

[34] Xing F-F, Chiu K-Y, Deng C-W, Ye H-Y, Sun L-L, Su Y-X, Cai H-J, Lo S-F, Rong L, Chen J-L, Cheng V-C, Lung DC, Sridhar S, Chan J-W, Hung I-N, Yuen K-Y. 2024. Post-COVID-19 pandemic rebound of macrolide-resistant Mycoplasma pneumoniae infection: a descriptive study. Antibiotics (Basel) 13:262. doi:10.3390/antibiotics13030262 38534697 PMC10967482

[35] Abushahin A, Toma H, Alnaimi A, Abu-Hasan M, Alneirab A, Alzoubi H, Belavendra A, Janahi I. 2024. Impact of COVID-19 pandemic restrictions and subsequent relaxation on the prevalence of respiratory virus hospitalizations in children. BMC Pediatr 24:91. doi:10.1186/s12887-024-04566-9 38302912 PMC10835825

[36] Teirlinck AC, Johannesen CK, Broberg EK, Penttinen P, Campbell H, Nair H, Reeves RM, Bøås H, Brytting M, Cai W, et al.. 2023. New perspectives on respiratory syncytial virus surveillance at the national level: lessons from the COVID-19 pandemic. EUR RESPIR J 61:2201569. doi:10.1183/13993003.01569-2022 37012081 PMC10069872

[37] Çağlar HT, Pekcan S, Yılmaz Aİ, Ünal G, Ercan F, Savaş S, Akcan ÖM, Ünsaçar MZ, Ünsaçar K, Özdemir M. 2023. The epidemiologic trend of respiratory syncytial virus has returned strongly to its origin after the pandemic: five-year data from a single center. PEDIATR Pulmonol 58:3582–3587. doi:10.1002/ppul.26696 37737535

[38] Xu D, Chen Z, Zhu G. 2024. Change of epidemiological characteristics of four respiratory viral infections in children before and during COVID-19 pandemic. Infect Dis Now 54:104858. doi:10.1016/j.idnow.2024.104858 38309644

[39] Kim HM, Rhee JE, Lee N-J, Woo SH, Park AK, Lee J, Yoo CK, Kim E-J. 2022. Recent increase in the detection of human parainfluenza virus during the coronavirus disease-2019 pandemic in the Republic of Korea. Virol J 19:215. doi:10.1186/s12985-022-01938-4 36510212 PMC9744062

[40] Li Y, Liang Y, Tang J, Li N, Yang Y, Guo W, Lin C, Wu J, Lin Y, Chen Q. 2023. Clinical impact of human parainfluenza virus infections before and during the COVID-19 pandemic in Southern China. MICROBES INFECT 25:105219. doi:10.1016/j.micinf.2023.105219 37734534

[41] Hong S, Li D, Wei Y, Zheng Y, Cai J, Zheng H, Zhang X, Deng Y, Han D, Wang J, Chen L, Li S, Qiu W, Ren M, Zou L. 2023. Epidemiology of respiratory pathogens in patients with acute respiratory tract infection in Xiamen, China: a retrospective survey from 2020 to 2022. Heliyon 9:e22302. doi:10.1016/j.heliyon.2023.e22302 38053876 PMC10694312

[42] Agrupis KA, Villanueva AMG, Sayo AR, Lazaro J, Han SM, Celis AC, Suzuki S, Uichanco AC, Sagurit J, Solante R, Yoshida L-M, Ariyoshi K, Smith C. 2021. If not COVID-19 what is it? Analysis of COVID-19 versus common respiratory viruses among symptomatic health care workers in a tertiary infectious disease referral hospital in Manila, Philippines. Trop Med Infect Dis 6:39. doi:10.3390/tropicalmed6010039 33808524 PMC8005933

[43] Champredon D, Bancej C, Lee L, Buckrell S. 2022. Implications of the unexpected persistence of human rhinovirus/enterovirus during the COVID-19 pandemic in Canada. INFLUENZA Other Respir Viruses 16:190–192. doi:10.1111/irv.12930 34747155 PMC8652650

[44] Madewell ZJ, Wang L-P, Dean NE, Zhang H-Y, Wang Y-F, Zhang X-A, Liu W, Yang W-Z, Longini IM, Gao GF, Li Z-J, Fang L-Q, Yang Y, Chinese centers for disease control and prevention (CDC) etiology of respiratory infection surveillance study team. 2023. Interactions among acute respiratory viruses in Beijing, Chongqing, Guangzhou, and Shanghai, China, 2009-2019. INFLUENZA OTHER Respir Viruses 17:e13212. doi:10.1111/irv.13212 37964991 PMC10640964

[45] Wu A, Mihaylova VT, Landry ML, Foxman EF. 2020. Interference between rhinovirus and influenza A virus: a clinical data analysis and experimental infection study. Lancet Microbe 1:e254–e262. doi:10.1016/s2666-5247(20)30114-2 33103132 PMC7580833

[46] Pan Q, Chen X, Yu Y, Zang G, Tang Z. 2024. The outbreak of seasonal influenza after the COVID‑19 pandemic in China: unraveling the "Immunity debt" Infect Dis Now 54:104834. doi:10.1016/j.idnow.2023.104834 37972819

[47] Liu B. 2017. Universal PCR primers are critical for direct sequencing-based enterovirus genotyping. J Clin Microbiol 55:339–340. doi:10.1128/JCM.01801-16 28031445 PMC5228251

[48] Liu B, Forman M, Valsamakis A. 2019. Optimization and evaluation of a novel real-time RT-PCR test for detection of parechovirus in cerebrospinal fluid. J Virol Methods 272:113690. doi:10.1016/j.jviromet.2019.113690 31283959

