# Supplementary material for: Epidemiology of respiratory pathogens in patients with acute respiratory infections during the COVID‐19 pandemic and after easing of COVID‐19 restrictions
Source: Microbiol Spectr. 2024 Sep 25;12(11):e01161-24. doi: 10.1128/spectrum.01161-24 (PMC11537120; doi:10.1128/spectrum.01161-24)
Supplement: Tables S1 and S2 — Table S1: Co-infection detected in patients with ARIs. Table S2: Total number and positive number (positivity rate) of ARI patients. [file spectrum.01161-24-s0001.docx]

Table S1 Co-infection detected in patients with ARTIs

|  | |  | Single‐infection | Double‐infection | Triple‐infection | Total |
| --- | --- | --- | --- | --- | --- | --- |
| Total | | Patients | 1917(91.50) | 165(7.88) | 13(0.62) | 2095 |
|  |  | Pathogens | 1917(83.86) | 330(14.44) | 39(1.71) | 2286 |
| Year | 2021 | Patients | 311(95.40) | 14(4.29) | 1(0.31) | 326 |
|  |  | Pathogens | 311(90.93) | 28(8.19) | 3(0.88) | 342 |
|  | 2022 | Patients | 266(93.99) | 16(5.65) | 1(0.35) | 283 |
|  |  | Pathogens | 266(88.37) | 32(10.63) | 3(1.00) | 301 |
|  | 2023 | Patients | 1340(90.17) | 135(9.08) | 11(0.74) | 1486 |
|  |  | Pathogens | 1340(81.56) | 270(16.43) | 33(2.01) | 1643 |
| Pathogens | | FluA | 446(19.51) | 52(2.27 ) | 4( 0.17) | 502(21.96) |
|  |  | FluB | 62( 2.71) | 8(0.35) | 0(0 ) | 70(3.06) |
|  |  | PIV | 129(5.64) | 19(0.83 ) | 6(0.26 ) | 154(6.74) |
|  |  | ADV | 148(6.47) | 58(2.54 ) | 7( 0.31) | 213(9.32) |
|  |  | RVs | 398(17.41 ) | 67( 2.93) | 6(0.26) | 471(20.6) |
|  |  | MPV | 138(6.04 ) | 22( 0.96) | 3(0.13 ) | 163(7.13) |
|  |  | RSV | 133( 5.82) | 17( 0.74) | 4(0.17 ) | 154(6.74) |
|  |  | CoV | 48(2.1) | 21(0.92) | 1(0.04 ) | 70(3.06) |
|  |  | MP | 369(16.14) | 51(2.23 ) | 6( 0.26) | 426(18.64) |
|  |  | Chlamydia | 9( 0.39) | 3(0.13 ) | 1(0.04 ) | 13(0.57) |
|  |  | BoV | 37(1.62 ) | 12(0.52 ) | 1( 0.04) | 50(2.19) |

Values presented in this table are numbers and percentages(%).

Abbreviations: FluA, influenza A; FluB, influenza B; PIV, parainfluenza virus; ADV, adenovirus; RVs, rhinovirus; MPV, metapneumovirus; RSV, respiratory syncytial virus; CoV, coronavirus; MP, mycoplasma pneumonia; BoV, bocavirus;

Table S2 Total number and positive number (positive rate) of ARIs patients in each month from 2021 to 2023

| Demographic | | COVID-19 | | | | Post- COVID-19 | | Total | |
| --- | --- | --- | --- | --- | --- | --- | --- | --- | --- |
|  |  | 2021 | | 2022 | | 2023 | | 2021-2023 | |
|  |  | Total | Positive n(%) | Total | Positive n(%) | Total | Positive n(%) | Total | Positive n(%) |
| Month | Jan | 65 | 18(27.69) | 125 | 52(41.6) | 65 | 4(6.15) | 255 | 74(29.02) |
|  | Feb | 34 | 2(5.88) | 68 | 6(8.82) | 112 | 22(19.64) | 214 | 30(14.02) |
|  | Mar | 64 | 6(9.38) | 90 | 18(20) | 321 | 132(41.12) | 475 | 156(32.84) |
|  | Apr | 117 | 25(21.37) | 109 | 21(19.27) | 345 | 59(17.10) | 571 | 105(18.39) |
|  | May | 129 | 26(20.16) | 110 | 36(32.73) | 422 | 58(13.74) | 661 | 120(18.15) |
|  | Jun | 166 | 49(29.52) | 120 | 26(21.67) | 334 | 57(17.07) | 620 | 132(21.29) |
|  | Jul | 179 | 64(35.75) | 133 | 26(19.55) | 266 | 51(19.17) | 578 | 141(24.39) |
|  | Aug | 84 | 27(32.14) | 129 | 42(32.56) | 287 | 65(22.65) | 500 | 134(26.8) |
|  | Sept | 88 | 31(35.23) | 86 | 18(20.93) | 304 | 114(37.5) | 478 | 163(34.1) |
|  | Oct | 103 | 36(34.95) | 116 | 23(19.83) | 366 | 147(40.16) | 585 | 206(35.21) |
|  | Nov | 37 | 7(18.92) | 38 | 11(28.95) | 570 | 237(44.58) | 645 | 255(39.53) |
|  | Dec | 114 | 35(30.70) | 37 | 4(10.81) | 900 | 540(60.00) | 1051 | 579(55.09) |

Values presented in this table are numbers and positive rate(%).

Values presented in this table are numbers and percentages(%).

Abbreviations: FluA, influenza A; FluB, influenza B; PIV, parainfluenza virus; ADV, adenovirus; RVs, rhinovirus; MPV, metapneumovirus; RSV, respiratory syncytial virus; CoV, coronavirus; MP, mycoplasma pneumonia; BoV, bocavirus;
